# Rapid and label-free microfluidic neutrophil purification and phenotyping in diabetes mellitus

**DOI:** 10.1038/srep29410

**Published:** 2016-07-06

**Authors:** Han Wei Hou, Chayakorn Petchakup, Hui Min Tay, Zhi Yang Tam, Rinkoo Dalan, Daniel Ek Kwang Chew, King Ho Holden Li, Bernhard O. Boehm

**Affiliations:** 1 Lee Kong Chian School of Medicine, Nanyang Technological University, Singapore; 2 Mechanical and Aerospace Engineering, Nanyang Technological University, Singapore; 3 Endocrine and Diabetes, Tan Tock Seng Hospital, Singapore; 4 Imperial College London, UK

## Abstract

Advanced management of dysmetabolic syndromes such as diabetes will benefit from a timely mechanistic insight enabling personalized medicine approaches. Herein, we present a rapid microfluidic neutrophil sorting and functional phenotyping strategy for type 2 diabetes mellitus (T2DM) patients using small blood volumes (fingerprick ~100 μL). The developed inertial microfluidics technology enables single-step neutrophil isolation (>90% purity) without immuno-labeling and sorted neutrophils are used to characterize their rolling behavior on E-selectin, a critical step in leukocyte recruitment during inflammation. The integrated microfluidics testing methodology facilitates high throughput single-cell quantification of neutrophil rolling to detect subtle differences in speed distribution. Higher rolling speed was observed in T2DM patients (*P* < 0.01) which strongly correlated with neutrophil activation, rolling ligand P-selectin glycoprotein ligand 1 (PSGL-1) expression, as well as established cardiovascular risk factors (cholesterol, high-sensitive C-reactive protein (CRP) and HbA1c). Rolling phenotype can be modulated by common disease risk modifiers (metformin and pravastatin). Receiver operating characteristics (ROC) and principal component analysis (PCA) revealed neutrophil rolling as an important functional phenotype in T2DM diagnostics. These results suggest a new point-of-care testing methodology, and neutrophil rolling speed as a functional biomarker for rapid profiling of dysmetabolic subjects in clinical and patient-oriented settings.

Diabetes mellitus (DM) is a prototypic dysmetabolic syndrome characterized by chronic hyperglycemia and remains a serious health burden globally with a predicted rise to 360 million by 2030^1^. In type 2 diabetes mellitus (T2DM), patients suffer from impaired glucose metabolism, which results in low-grade inflammation and activation of the innate immune system, with an increased risk of cardiovascular complications^2^,^3^,^4^,^5^. While differential leukocyte count and C-reactive protein (CRP) level are routinely measured and associated with cardiovascular mortality^6^,^7^, macro/microvascular complications^8^,^9^ and metabolic phenotypes^10^ in diabetic patients, it is imperative to develop new cell-based biomarkers that can quantify specific immune functions in addition to leukocyte enumeration. Neutrophils, the key effector cells of the innate immune system, are known to play a pivotal role in T2DM pathogenesis as well as its associated vascular complications^11^. Various neutrophil dysfunctions have been reported in T2DM patients including cell stiffening^12^,^13^, impaired chemotaxis^14^,^15^ and phagocytosis^16^ resulting in an increased susceptibility to bacterial infections^17^. A comprehensive phenotyping of neutrophil functions in T2DM patients therefore enables early and direct characterization of immune health for timely therapeutic interventions. However, it is non-trivial to isolate neutrophils in their native state from peripheral blood as conventional neutrophil isolation methods are laborious and prone to cell activation^18^, which can be greatly minimized with antibodies-free neutrophil isolation methods^19^. Critically, there are currently no point-of-care (POC) technologies enabling the quantification of neutrophil function related to a low grade inflammatory state. This advocates a strong clinical need to develop novel technologies for rapid, label-free neutrophil sorting and functional phenotyping to better characterize the inflammatory status of T2DM patients.

With advances in microfabrication, microfluidics provides an exciting tool box for POC diagnostics and biomedical research with its low consumption of sample and reagents, device miniaturization, and high throughput single-cell analysis^20^. Several microfluidics technologies have been developed for neutrophil sorting^21^,^22^,^23^ and are used to study inflammatory responses in patients with severe trauma and burn injury^21^, as well as asthma^22^. These devices achieve neutrophil purification based on antibodies binding (CD66b and P-selectin), and subsequent characterizations are performed on chip as the sorted neutrophils are attached inside the microchannel. This inherently limits downstream applications including *in vitro* cell culture assays and flow cytometry analysis since it is challenging to recover the sorted neutrophils from the device. Noteworthy, no published reports to date have applied microfluidics for neutrophil functional phenotyping in T2DM patients, which would be invaluable in studying the association among alterations of cardiovascular risk factors, abnormal leukocyte phenotypes and the accompanied endothelial dysfunctions.

In this work, we develop a novel microfluidic technology for rapid, non-perturbing isolation of neutrophils from small blood volumes (fingerprick) in a single-step and label-free manner. The purified neutrophils are used to characterize their rolling behavior on E-selectin, a critical step in leukocyte recruitment during inflammation^24^,^25^ using microfluidics assay integrated with automated, high throughput single-cell measurement. The combined microfluidic cell sorting and functional phenotyping strategy was clinically validated using blood samples from healthy subjects and T2DM patients, which revealed a significant difference in the neutrophil rolling pattern between the two groups. Higher neutrophil rolling speed was observed in T2DM patients (*P* < 0.01), which strongly correlated with neutrophil intracellular reactive oxygen species (ROS) level, rolling ligand P-selectin glycoprotein ligand 1 (PSGL-1) expression, as well as established cardiovascular risk factors (cholesterol, high-sensitive C-reactive protein (CRP) and HbA1c). These results suggest a new methodology and surrogate biomarker for rapid neutrophil functional phenotyping (total time <20 min), and can be further developed into POC testing methods for real-time risk stratification, and timely monitoring of risk modifying therapeutics in patients with a dysmetabolic state such as diabetes mellitus.

## Results

### Microfluidic neutrophil purification using Dean Flow Fractionation (DFF)

**Figure 1:**
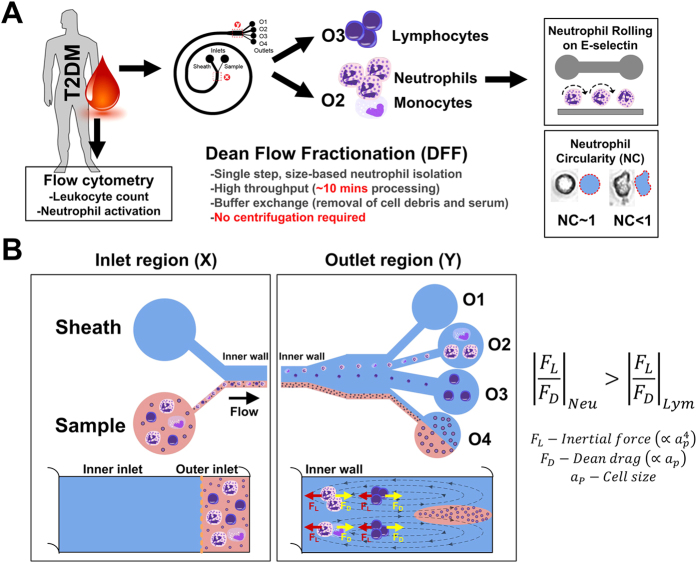
Single step and label-free neutrophil sorting using Dean Flow Fractionation (DFF) microfluidic technology. (**A**) Experimental workflow for neutrophil isolation and phenotyping in T2DM patients. Blood samples are lysed and processed using the 2-inlet, 4-outlet spiral microdevice for efficient size-based neutrophil sorting. The purified neutrophils are used for *in vitro* cell rolling assay in a microchannel functionalized with E-selectin, as well as shape measurement (neutrophil circularity). (**B**) Schematic illustration of DFF separation principle. Under the influence of Dean vortices, small cellular constituents (platelets and lysed RBCs) and free haemoglobin migrate laterally towards inner wall and back to outer wall due to Dean drag forces (F_D_ (yellow arrows)). Larger leukocytes experience additional strong inertial lift forces (F_L_ (red arrows)) and due to the strong dependence of F_L_ and F_D_ on cell size, larger neutrophils/monocytes (10–12 μm) focus closer to the inner wall and are sorted into outlet 2 while smaller lymphocytes (~7–8 μm) are collected at outlet 3. Outlet 4 is used for removal of platelets, lysed RBCs and free haemoglobin.

We have previously developed a novel microfluidic cell sorting technology termed as Dean Flow Fractionation (DFF) for size-based separation of diseased cells including circulating tumor cells (CTCs)^26^ and microorganisms^27^ from whole blood. DFF is an inertial microfluidics based sorting method which involves the lateral migration of particles or cells across streamlines to focus at distinct positions due to dominant inertial forces (F_L_) at high flow rates (Reynolds number, Re ~50–100)^28^. In DFF systems, fluid flowing through a spiral microchannel experiences centrifugal acceleration directed radially outward, leading to the formation of two symmetrical counter-rotating Dean vortices at the top and bottom halves of the channel^29^. The Dean vortices impose additional lateral Dean drag force (F_D_) on the cells/particles and both forces (F_L_ and F_D_) scale non-linearly with size to achieve differential focusing and separation of cells/particles of different sizes^30^,^31^,^32^. Herein, we exploited the subtle cell size difference between leukocyte subtypes and developed a novel 4-outlet DFF spiral device to purify neutrophils from lysed whole blood in a single-step manner (Fig. 1). More importantly, the technology enables simultaneous buffer exchange^33^ and the sorted neutrophils are continuously washed on chip by the removal of lysed RBCs and lysis buffer prior collection.

The DFF spiral device was fabricated in polydimethylsiloxane (PDMS) and consists of a two-inlet, four-outlet spiral microchannel (500 μm (w) × 115 μm (h)) with a total length of ~10 cm. The channel height was fixed at 115 μm so that only the larger leukocytes (~8 to 12 μm, a_p_/h > 0.07, where a_p_ is particle size) can experience inertial focusing and equilibrate near the inner wall. Near the outlet region, the channel gradually expands to a larger width (1000 μm) at the bifurcation with 4 collection outlets of different widths (starting from inner wall: outlet 1 (O1): 100 μm, outlet 2 (O2): 150 μm, outlet 3 (O3): 400 μm, outlet 4 (O4): 350 μm). To purify neutrophils from whole blood, human whole blood was lysed with RBCs lysis buffer (1:10 volume) followed by quenching with 1× phosphate-buffered saline (PBS) supplemented with 0.5% bovine serum albumin (BSA) (1:2 volume). The diluted lysed blood sample was pumped into the outer inlet at 130 μLmin^−1^ and sheath fluid (1× PBS supplemented with 0.1% BSA) was pumped through the inner inlet at a higher flow rate (10×, 1300 μLmin^−1^) to confine the sample stream near the outer wall (Fig. S1). As blood sample flows along the channel, cells undergo lateral migration towards the inner wall due to dominant F_D_. The smaller platelets and lysed RBCs (a_p_/h < 0.05) recirculate back to outer wall to complete a Dean cycle (DC 1) while larger leukocytes (a_p_/h > 0.07) experience additional inertial lift forces (F_L_) and focus near the inner wall. Due to cell size differences, the larger neutrophils/monocytes (10–12 μm, a_p_/h~0.9-0.1) experience stronger F_L_ (towards inner wall direction) than lymphocytes (~7–8 μm, a_p_/h ~0.07) and equilibrate closer to inner wall, resulting in leukocyte fractionation of neutrophils and lymphocytes into outlet 2 and 3, respectively (Fig. 1B). This facilitates efficient neutrophil purification as the sorted neutrophils are resuspended in sheath buffer during collection while the original lysed blood sample (platelets, lysed RBCs and free hemoglobin) is eluted at outlet 4 (Fig. S1).

### Characterization of direct neutrophil sorting from whole blood (fingerprick)

**Figure 2:**
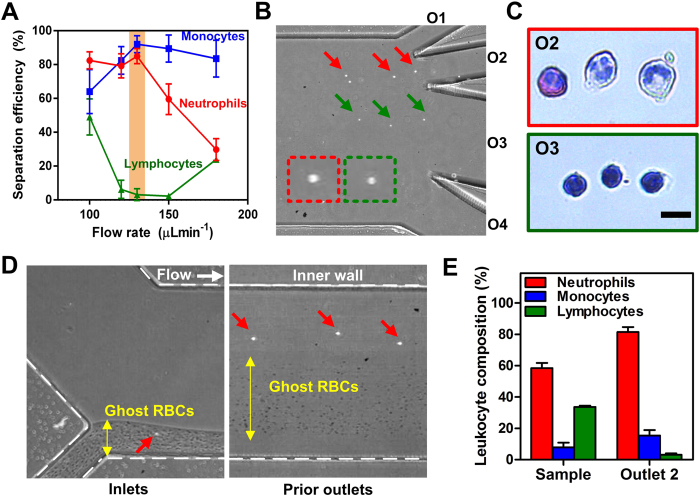
Neutrophil purification using DFF microdevice. (**A**) Flow rate characterization on neutrophil sorting in outlet 2 of spiral device. Optimal separation was achieved at 130 μLmin^−1^ (highlighted). Mean ± s.d. from *n* = 2–4. (**B**) Representative high speed images showing separation of larger neutrophils (red arrows) and smaller lymphocytes (green arrows) into different outlets. Enlarged inset images (dotted boxes) illustrate the cell size difference. (**C**) Wright-Giemsa staining of sorted neutrophils from outlet 2 (red box) and lymphocytes from outlet 3 (green box). (**D**) Representative high speed images of lysed blood processing and separation of ghost RBCs (yellow arrows) and leukocytes (red arrows). (**E**) Leukocyte composition of inlet sample and outlet 2 post DFF sorting. Mean ± s.d. from *n* = 3.

To determine the optimal flow conditions for neutrophil purification, blood samples were lysed and washed with PBS to remove RBCs contaminants. The washed samples consisting mainly of leukocytes and platelets were then pumped into the spiral DFF device at different flow rates and eluents were collected from the 4 outlets for flow cytometry analysis. For leukocyte differential analysis, leukocytes were gated based on forward and side scatter signals, and stained with a cocktail of antibodies to identify neutrophils, monocytes and lymphocytes (see methods and Fig. S1). As shown on Fig. 2A, the larger neutrophils and monocytes were efficiently sorted (>80%) into outlet 2 at a sample flow rate of 120–130 μLmin^−1^ and majority of the smaller lymphocytes (~95%) were separated into outlet 3. This was evident by high speed microscopic image showing inertial focusing of larger cells closer to the inner wall (Fig. 2B). The sorted leukocytes were also stained with Giemsa Wright stain to confirm neutrophil identity at outlet 2 and the cells were shown to well preserve their morphology post separation (Fig. 2C). As the flow rate increased above 130 μLmin^−1^, neutrophil recovery into outlet 2 was reduced while monocytes sorting remained unaffected. Hence, the optimal flow rate was set at 130 μLmin^−1^ for subsequent experiments to achieve high throughput neutrophil sorting with minimal lymphocytes contamination in outlet 2. This single-step procedure requires ~10 min to isolate neutrophils from a drop of fingerprick blood (~50 μL of whole blood; total lysed blood volume ~1–1.5 mL), which is significantly faster than existing neutrophil isolation methods that are laborious and require large blood volumes (~3–10 mL). Besides neutrophil purification, we also demonstrated label-free fractionation of monocytes and lymphocytes using peripheral blood mononuclear cells (PBMCs) obtained from density gradient centrifugation (Fig. S2). With the ability to process high cell concentrations (~10^7 ^cells/mL)^33^, this high throughput, size-based monocyte purification approach is a major improvement over current strategies based on CD14 + affinity selection which can affect cytokine production^34^. For POC testing, it is important to minimize manual sample preparation to ensure results consistency. We next studied the effects of direct lysed blood processing (without centrifugation) using DFF and demonstrated similar neutrophil separation performance using lysed blood samples quenched with twice the saline volume (Fig. S3). Neutrophils were focusing near the inner wall and clearly separated from the ghost RBCs (Fig. 2D) to achieve high neutrophil purity (~90%) in outlet 2 (Fig. 2E). Lastly, we characterized neutrophil CD66b expression and intracellular ROS level before and after DFF separation and showed that ROS level of sorted untreated neutrophils (obtained from healthy subjects) remained significantly lower than activated (PMA-treated) neutrophils (Fig. S3). Consistent with previous studies^32^, this indicates that the high flow conditions (~m/sec) within the spiral device have negligible effects on leukocyte activation and further validates the use of DFF neutrophil purification technology for processing blood samples from patients with T2DM or other states of dysmetabolism (e.g. cardiovascular diseases without diabetes).

### Single cell analysis of neutrophil rolling on E-selectin

**Figure 3:**
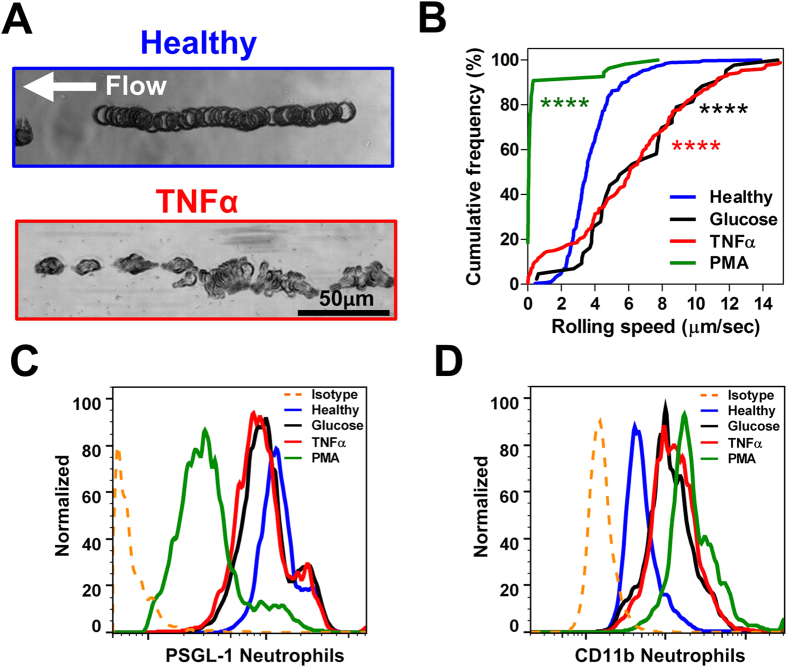
Neutrophil rolling on E-selectin. (**A**) Image overlay (60× magnification) from captured cell rolling videos highlight distinct differences in rolling trajectories between healthy and TNF-α-treated neutrophils. (**B**) Cumulative frequency curves of neutrophil rolling speed for healthy, glucose-treated (30 mM), TNF-α-treated (10 ng/mL) and PMA-treated (1 μM) neutrophils. The rolling speeds for all treated groups (~50–200 cells in each group) were significantly different as compared to healthy neutrophils (*P* < 0.0001). Representative flow cytometric analysis of (**C**) PSGL-1 and (**D**) CD11b expression on healthy and treated neutrophils. Cells were incubated with mouse APC anti-human PSGL-1 IgG2a/mouse APC IgG2a isotype and FITC mouse anti-human CD11b (activated) IgG1/FITC mouse IgG1 isotype.

To study neutrophil rolling phenotype on E-selectin, we coated a straight microchannel (1 cm (l) × 400 μm (w) × 60 μm (h)) with recombinant human E-selectin (50 μg/mL) to simulate flow conditions in post capillary venules (~1–10 dynecm^−2^). DFF-sorted neutrophils were pumped into the channel at physiological shear conditions (2 dynecm^−2^) and timelapse imaging was used to capture neutrophil rolling. A MATLAB tracking algorithm was developed to track neutrophil rolling trajectories and speed measurement for high throughput single cell analysis. As shown on Fig. 3A, healthy neutrophils exhibited steady rolling behavior in a straight path while rolling trajectories for tumor necrosis factor alpha (TNF-α)-activated neutrophils were discontinuous with “flipping” motions. Rolling speeds of glucose-treated (30 mM) and TNF-α-activated (10 ng/mL) neutrophils were higher than healthy neutrophils (*P* < 0.005) and PMA-treated (1 μM) neutrophils did not roll, but instead adhered firmly on E-selectin under flow (Fig. 3B). We further investigated these differences in rolling phenotypes by measuring neutrophil PSGL-1 expression, which is known to mediate leukocyte rolling on E-selectin^24^,^35^, as well as CD11b, a Mac-1 integrin expressed on leukocytes that supports adhesion to E-selectin^36^. PSGL-1 expression on neutrophils decreased with both glucose and TNF-α treatments and the downregulation was more significant for PMA-treated neutrophils, which is consistent with previous report showing PSGL-1 shedding in activated leukocytes^37^ (Fig. 3C). Similarly, neutrophil CD11b level was upregulated for all treated conditions, with a higher increase in PMA-treated neutrophils (Fig. 3D). These results confirmed that altered surface expression of PSGL-1 and CD11b affect neutrophil rolling on E-selectin and suggest the use of cell rolling phenotype as a novel functional biomarker to assess neutrophil activation in diabetes patients.

### Neutrophil immunophenotyping in T2DM patients

**Figure 4:**
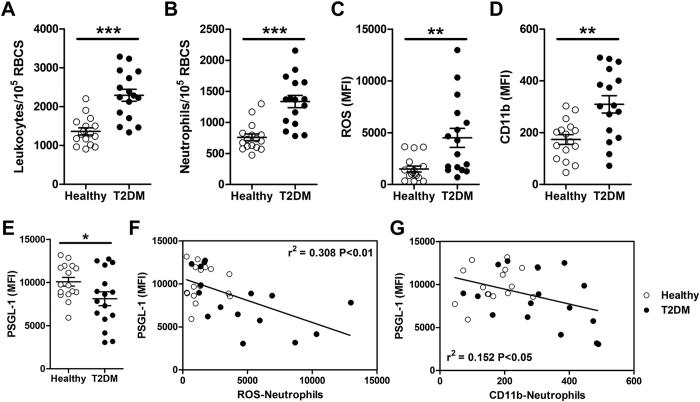
Neutrophil immunophenotyping in T2DM patients. Flow cytometry analysis on (**A**) leukocyte and (**B**) neutrophil count, and expression of (**C**) intracellular reactive oxygen species (ROS), (**D**) CD11b and (**E**) PSGL-1 on neutrophils from healthy (*n* *=* *16*) and T2DM patients (*n* *=* *16*). Data are presented as mean ± s.e.m. *P < 0.05, **P < 0.01 and ***P < 0.001. Correlation of neutrophil PSGL-1 level with (**F**) ROS and (**G**) CD11b expression.

As neutrophil dysfunctions have been reported previously in T2DM patients^15^,^16^,^38^, we first performed immunophenotyping in healthy subjects (n = 16) and T2DM (n = 16) patients using flow cytometry and C-reactive protein (CRP) measurement, a key marker for low-grade inflammatory state and cardiovascular risk (summarized in Table 1). Both leukocyte and neutrophil counts were higher in T2DM patients as compared to healthy subjects (*P* < 0.001), indicating increased inflammatory responses *in vivo* (Fig. 4A,B). Average neutrophil ROS level (Fig. 4C) and CD11b expression (Fig. 4D) were also elevated in T2DM group (*P* < 0.01), thus confirming more pronounced neutrophil activation in T2DM patients. Interestingly, there was a significant downregulation of neutrophil PGSL-1 expression in T2DM patients (*P* < 0.05) (Fig. 4E). This was well correlated with neutrophil ROS level (Fig. 4F) and CD11b expression (Fig. 4G) in both healthy and T2DM patients as lower PSGL-1 expression was associated with increasing neutrophil activation. Negligible differences in PSGL-1 and CD11b expression were observed between DFF-sorted neutrophils and neutrophils washed with centrifugation in T2DM patients (Fig. S4), further validating DFF as an efficient neutrophil purification technology for downstream assays or POC testing. Besides neutrophils, we also observed downregulation of PSGL-1 expression in monocytes of T2DM patients (*P* < 0.01) which warrants further studies to provide insights in monocyte-endothelial interactions and cardiovascular complications in T2DM patients (Fig. S5). Taken together, these results clearly indicate proinflammatory situation in T2DM patients with the increased presence of activated neutrophils which can contribute to chronic inflammatory conditions^39^.

### Distinct neutrophil rolling phenotype in T2DM patients

**Figure 5:**
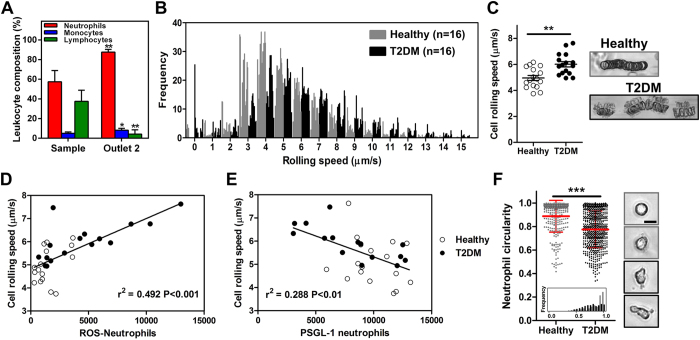
Neutrophil rolling in T2DM patients. (**A**) High purity of DFF-sorted neutrophils (~90%) from T2DM patients. Mean ± s.d. from *n* = 6 *P < 0.05, **P < 0.01. (**B**) Frequency distribution and (**C**) average rolling speed of neutrophils from healthy (*n* = *16*) and T2DM patients (*n* = *16*) in microchannel functionalized with E-selectin. Mean ± s.e.m. *P < 0.01. Representative images overlay of neutrophil rolling trajectories in healthy and T2DM patients. Correlation of average rolling speed with neutrophil (**D**) intracellular ROS level and (**E**) PSGL-1 expression. (**F**) Neutrophil circularity of healthy and T2DM patients. Mean ± s.d. *n* = 300 cells (from 5 healthy controls) and 500 cells (from 7 T2DM patients). ***P < 0.001. Inset plot shows frequency distribution of neutrophil circularity. Representative brightfield images (60× magnification) illustrating neutrophil shape differences from healthy (grey box) and T2DM (black box) patients.

Microfluidic rolling assays have been reported previously^40^,^41^,^42^, but are limited by design complexity and conventional leukocyte isolation methods prior assay. To characterize neutrophil rolling phenotype in T2DM patients, we isolated neutrophils from healthy subjects (n = 16) and T2DM (n = 16) patients using the developed DFF microfluidic technology and measured their rolling speed on E-selectin in a microfluidic assay. As expected, high neutrophil purity (~90%) was achieved in outlet 2 of the spiral device when processing blood samples from T2DM patients (Fig. 5A). Neutrophil rolling speed varied between healthy and T2DM patients as evident by a shift towards higher rolling speed frequency distribution for T2DM patients (Fig. 5B). This gave rise to higher average rolling speed in T2DM patients (6.01 ± 0.21 μm/sec (T2DM) vs. 4.95 ± 0.19 μm/sec (healthy) *P* < 0.01) and the rolling trajectories of T2DM neutrophils were more discontinuous and irregular as compared to healthy neutrophils (Fig. 5C). Average rolling speed was strongly correlated to neutrophil ROS level (Fig. 5D) and PSGL-1 expression (*P* < 0.001) (Fig. 5E) but not CD11b (Fig. S6) in both healthy subjects and T2DM patients, suggesting a key role of PSGL-1 in mediating leukocyte rolling during inflammation. Although the age group differs between healthy subjects and T2DM patients, there was no correlation between patient age with CRP level and average rolling speed (Fig. S7), thus indicating that the observed changes in neutrophil rolling phenotype was due to factors other than age-related inflammation. This is further validated when we compared a subset of healthy and T2DM patients without inflammation (CRP < 1 mg/mL) and showed that the rolling speed remained higher in T2DM patients (Fig. S7). We also compared neutrophil rolling pattern with blood samples obtained using venipuncture or fingerprick from the same patient. Negligible differences in neutrophil rolling speed were observed between different blood sampling methods (*P* ~0.35–0.68), indicating the robustness of the developed microfluidic strategies used for neutrophil sorting and functional phenotyping (Fig. S8). Lastly, we characterized neutrophil morphology based on the neutrophil circularity (NC) index. As shown on Fig. 5F, DFF-sorted healthy neutrophils were mostly circular (~76% of cells with NC > 0.85) while neutrophils from T2DM patients were more heterogeneous in shape, with a higher number of elongated cells (~55% of cells with NC 0.5~0.8) present. We hypothesize that such cytoplasmic prolongations are caused by neutrophil activation which is known to affect cell morphology^43^. In summary, the DFF neutrophil purification technology was successfully validated in a cohort of healthy subjects and T2DM patients and we provided clear evidence that healthy and diabetic neutrophils possess distinct activation profiles and cell morphologies which affect their rolling phenotype on E-selectin.

### Neutrophil rolling as a functional biomarker for T2DM testing

**Figure 6:**
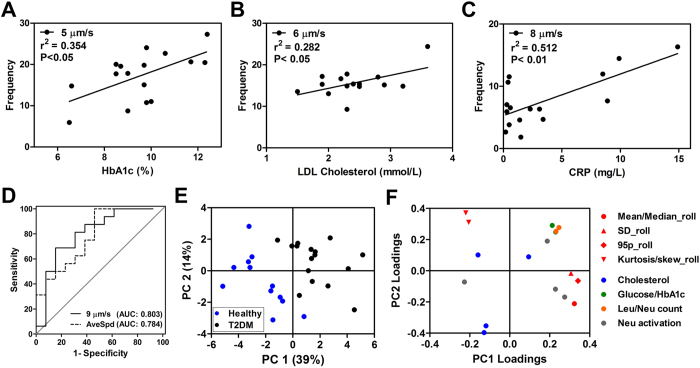
Neutrophil rolling phenotype as a functional biomarker for T2DM testing. Correlation of specific rolling speed frequency with (**A**) HbA1c (%), (**B**) LDL cholesterol and (**C**) C-reactive protein (CRP). (**D**) ROC analysis of patient data using average rolling speed and rolling speed frequency at 9 μm/sec. (**E**) PCA score plot shows well separation of healthy and T2DM patients on the 1^st^ principle component. (**F**) Loadings plot shows the contribution of each metric to the 1^st^ and 2^nd^ principle component score values.

After having established that neutrophils from T2DM patients rolled faster on E-selectin as compared to healthy subjects, we sought to determine if these functional differences can be associated to their clinical conditions by analyzing rolling speed frequencies in T2DM patients that are higher than the average rolling speed of healthy subjects (≥5 μm/sec). It is likely that patients with higher cardiovascular risk factors have faster rolling neutrophils due to increased level of low-grade inflammation. Indeed, higher hemoglobin A1c (HbA1c) level corresponded to higher frequency for rolling speed 5 μm/sec in T2DM patients (Fig. 6A), and low-density lipoprotein (LDL) cholesterol and CRP levels were also associated with increasing frequency of faster rolling neutrophils at 6 and 8 μm/sec, respectively (Fig. 6B,C). These relationships clearly illustrate the importance of glucose and cholesterol metabolism in neutrophil activation^44^ which can affect neutrophil functionality (cell rolling) and thus attenuate leukocyte recruitment/response to inflamed endothelium. To further assess the clinical efficacy of neutrophil rolling speed as a functional biomarker in diabetes testing, we showed that the rolling speed frequency (5 μm/sec) was a more sensitive indicator for HbA1c (%) than fasting glucose level in T2DM patients (Fig. S9). Analysis of the receiver operating characteristics (ROC) of the patient data using a diagnostics cutoff of rolling speed frequency at 9 μm/sec yields a sensitivity and specificity of ~81% and ~70%, respectively (Fig. 6D). This is comparable to a recent microfluidic device developed to characterize neutrophil chemotaxis for asthma diagnostics^22^. Principal component analysis (PCA) on clinical measurements (see Table 1) and rolling speed distributions was next performed which indicated well separation of healthy and T2DM patients by the 1^st^ principle component score (Fig. 6E). Multiple parameters including mean, median (Mean/Median_roll), standard deviation (SD_roll), skewness and kurtosis (Kurtosis/skew_roll) were used to describe the distribution characteristic of the neutrophils rolling speed. Rolling speeds greater than 95 percentile were averaged (95p_roll) and used to capture distribution on the right tail. In addition, PCA analysis also revealed that rolling speed and its distribution characteristic have strong contributions in discerning diabetes phenotype, as judged by the large magnitude in the 1^st^ principle components loadings (Fig. 6F). Lastly, we studied the effects of established vascular risk modifying drugs including metformin and pravastatin on rolling phenotype of healthy neutrophils. These drugs are commonly used in diabetic patients as well as patients with a dysmetabolic state (i.e. pre-diabetes, CHD) and have additional anti-inflammatory effects that reduce the risk of developing diabetes^45^. They have been described to alter neutrophil chemotaxis and phagocytic activities^46^,^47^ and are thus likely to affect other migratory responses in neutrophil-endothelial interactions. To avoid prior exposure to these medications, we performed the experiments using healthy subjects and incubated the DFF-purified neutrophils with metformin (1 mM) and pravastatin (20 μM) for 1 h before microfluidic rolling assay. Average rolling speed of both drug-treated neutrophils were higher than controls (untreated) in all paired observations (*P* < 0.05), which suggests rapid and active alteration of neutrophil rolling phenotype and its potential as an inflammatory functional marker for drug monitoring (Fig. S10).

## Discussion

Neutrophil dysfunctions are widely implicated in T2DM pathogenesis, but at present, there is a critical lack of tools including POC technologies measuring neutrophil phenotypes to assess patient inflammatory status. Towards that end, we describe the development and characterization of a new microfluidic technology (DFF) for rapid isolation of neutrophils from whole blood in a single-step and label-free manner. A microfluidic assay combined with automated cell tracking is also developed to study the rolling phenotype of neutrophils on E-selectin, a key step in leukocyte recruitment during inflammation. Using this integrated microfluidic testing strategy, we demonstrated that neutrophils from healthy subjects and T2DM patients have distinct activation profiles and cell morphologies which affect their rolling behavior. Interestingly, neutrophil rolling speed is strongly correlated to its activation (intracellular ROS), PSGL-1 expression, as well as cardiovascular risk factors including CRP, cholesterol and HbA1c levels in T2DM patients. These intriguing results clearly suggest neutrophil rolling speed as a novel functional biomarker for low-grade inflammatory profiling in T2DM patients.

A significant breakthrough of our integrated microfluidic approach is the rapid isolation of neutrophil in its native state from whole blood without antibodies labeling. This is achieved by efficient buffer exchange of the sorted neutrophils which removes RBCs and serum contaminant without centrifugation. Eosinophils might be a potential contaminant as they have similar cell size as neutrophils and would get sorted into outlet 2. However, peripheral eosinophils are ~30-fold fewer than neutrophils with lower percentages found in T2DM patients^48^, and they have significantly less binding affinity to E-selectin^49^,^50^. As we measure ~300–500 cell rolling speeds per patient, presence of eosinophils is therefore unlikely to affect our functional assay. Secondly, neutrophils are phenotypically heterogeneous cell population and single-cell analysis is highly crucial for accurate assessment of immune functions in dysmetabolic individuals. By developing a cell tracking algorithm to automate rolling speed measurement, it greatly facilitates high throughout single-cell characterization of cell morphology and neutrophil rolling speed distribution to detect subtle anomalies between healthy subjects and T2DM patients.

The functional phenotype of circulating leukocytes and its impact on leukocyte-endothelial interactions in T2DM patients is currently not well understood. During endothelial inflammation, leukocyte recruitment is a multi-step process involving cell rolling, adhesion and transmigration through blood vessel walls to the site of injury. E-selectin is a cell adhesion molecule expressed on inflamed vessel walls to initiate leukocyte capture^25^ and is mediated by several sialyl Lewis^x^ presenting ligands expressed on leukocytes including PSGL-1, glycosylated CD44 and E-selectin ligand 1 (ESL-1)^24^. Under shear flow, E-selectin engagement can lead to L-selectin and PSGL-1 redistribution and co-localization into clusters on the trailing edge of human neutrophils^51^. As L-selectin expression in neutrophils of diabetes patients and control groups were reported to be similar^52^,^53^ and PSGL-1 expression decreases in PMA-activated leukocytes^37^ which can affect their rolling functionalities^54^,^55^, these prompted us to measure neutrophil PSGL-1 expression and its rolling phenotype on E-selectin in T2DM patients. It is well established that neutrophil dysfunction is associated with increased susceptibility to infection in diabetic patients^17^ and attenuated neutrophil recruitment can further contribute to this complication^11^. Indeed, our data support this hypothesis as we observe significant downregulation of neutrophil PSGL-1 expression which is associated with higher neutrophil rolling speed in T2DM patients. Consistent with previous observations that leukocyte rolling time is critical for firm adhesion in leukocyte recruitment^56^, the observed neutrophil phenotypic changes in T2DM patients can potentially impair initial neutrophil capture, PSGL-1 mediated signaling^57^, as well as cell arrest through β2 integrins^58^, thereby leading to defective neutrophil-endothelial interactions during inflammation.

Another important feature for POC diabetes testing is to monitor dynamic immune responses to drug treatment. As proof-of-feasibility, we demonstrated that common diabetic drugs such as pravastatin and metformin can affect neutrophil rolling phenotype. Although we did not observe significant changes in drug-treated neutrophil PSGL-1 expression (data not shown), pravastatin is known to affect another E-selectin rolling ligand CD44 expression in cancer cells by increasing CD44 shedding^59^. Similarly, metformin is shown to preferentially target CD44 + cancer stem cells for chemotherapy^60^. Therefore, it is likely that statin and metformin-treated neutrophils have altered CD44 expression with different rolling behavior on E-selectin. As future work, we plan to study other E-selectin rolling ligands in T2DM patients to identify key mediators and potential therapeutic drug candidates to restore neutrophil-endothelial interactions.

Lastly, it should be noted that we validated our developed microfluidics technologies in a cross sectional case control study and did not compare inter-ethnic differences or standardize patients for medications and lifestyle habits. Nevertheless, analysis of the typical clinical measurements (see Table 1) with neutrophils associated measurements using PCA revealed that healthy and T2DM patients are well separated on 1^st^ principle component scores. Neutrophils rolling metrics were found to have comparable loading values with typical clinical measurements (i.e. fasting glucose level and HbA1c) which suggests that neutrophils associated metrics are also important in defining diabetes phenotype. The neutrophil phenotypic information obtained in this pilot study would enable us to design a larger scale diabetic population study in future to validate our findings, and monitor cardiovascular events or complications among high-risk patients.

In summary, we have developed a new microfluidic approach to purify neutrophils from whole blood based on subtle cell size differences with other leukocyte subtypes without antibodies labeling. From our pilot study, neutrophil rolling speed on E-selectin emerges as a potential functional biomarker for inflammatory profiling in T2DM patients. Since our microfluidic system only requires small blood volumes (finger prick; ~100 μL) for neutrophil sorting and rolling assay, it can be further developed into point-of-care testing method for rapid inflammatory profiling and stratification of patients with diabetes or dysmetabolic syndrome.

## Methods

### Microdevice fabrication

The DFF spiral and straight channel microfluidic devices were fabricated in polydimethylsiloxane (PDMS) using standard soft lithography methods. *See Supplemental Information for more details.*

### Immunostaining and flow cytometry analysis

All antibodies were purchased from BioLegend and flow cytometry analysis was performed using BD LSR Fortessa flow cytometer (BD Biosciences). To determine total leukocyte count, whole blood was diluted (1:20) in 1× PBS supplemented with 0.1% BSA, and stained with FITC-labeled anti-CD45 (1:20) for 30 min at 4 °C directly. Leukocyte and neutrophil count were normalized based on 10^5^ RBCs count. For leukocyte differentiation analysis, whole blood was lysed with RBCs lysis buffer (eBioscience Inc.) (1:10 v/v) for 3 min at room temperature and quenched with 1× PBS supplemented with 0.5% BSA. The leukocyte sample was washed twice at 1000× g for 4 min and stained with a cocktail of antibodies including FITC-labeled anti-CD45 (1:20), FITC-labeled anti-CD3 (1:20), APC-labeled anti-CD19 (1:20), APC-labeled anti-CD66b (1:400), APC-labeled anti-P-selectin glycoprotein ligand-1 (PSGL-1) (1:20) and PE-labeled anti-CD14 (1:20) for 30 min at 4 °C, followed by washing before flow cytometry analysis. Leukocyte subtypes were gated based on forward and side scatter and identified as neutrophils (CD45 + CD66b+), monocytes (CD45 + CD14+) and lymphocytes (CD3 + CD19+). For characterization of neutrophil activation, whole blood was lysed and the enriched leukocytes were stained with FITC-labeled anti-CD45 (1:20), anti-FITC-labeled CD11b (1:20), and APC-labeled anti-CD66b (1:20) for 30 min at 4 °C. Non-specific antibody binding was also examined using corresponding FITC/APC-labeled isotype negative control antibodies. Intracellular reactive oxygen species (ROS) generation by neutrophils was determined by incubating the leukocytes with 2′-7′-dichlorodihydrofluorescein diacetate (10 μM) (DCFH-DA, Invitrogen) for 30 min at room temperature. Neutrophil expression of PSGL-1, CD11b and ROS were determined based on median fluorescence intensity (MFI). Positive control for neutrophil activation was measured based on phorbol myristate acetate (PMA, 2 nM, Sigma-Aldrich) treatment of neutrophils for 20 min at room temperature. To compare neutrophil activation between RBCs lysis and DFF microfluidics sorting, DFF-purified neutrophils were stained using the described protocol.

### Microfluidic DFF device operation

For flow rate characterization, leukocytes enriched using RBC lysis method were washed by centrifugation (described previously) and loaded into a 3-mL syringe for device perfusion. For direct lysed blood processing and clinical testing, whole blood was lysed and quenched with 1× PBS supplemented with 0.5% BSA prior use. Leukocyte sample and sheath buffer (1× PBS with 0.1% BSA) were pumped into the 2-inlet, 4-outlet spiral microfluidic device at an optimized flow rate of 130 μLmin^−1^ and 1300 μLmin^−1^ respectively (ratio of 1:10) by separate syringe pumps (Chemyx Inc.). The DFF device was allowed to run for 2 min to stabilize the flow before collecting the eluent from outlet 2 (purified neutrophils). An inverted phase-contrast microscope (Nikon Eclipse Ti) equipped with a high-speed camera (Phantom v9.1) was used to capture phase-contrast bright-field images during the device operation.

### Neutrophil rolling characterisation

Briefly, a straight microchannel (1 cm length by 400 μm width by 60 μm height) was coated with E-selectin (50 μg/mL, Peprotech) for 1 h at 4 °C and blocked with 0.5% BSA in PBS for 30 min at room temperature. After blocking, the channel was connected to a syringe loaded with 0.1% BSA in PBS to prime and wash away excess E-selectin in the channel. For rolling assay, CaCl_2_ (20 μM, Sigma-Aldrich) was added to the DFF-purified neutrophils to facilitate the calcium-dependent interactions of neutrophil binding and rolling on E-selectin. ~10–20 μL of DFF-purified neutrophils (~10^6 ^cells/mL) was loaded at the inlet reservoir and the syringe was set to withdraw at 2.6 μL/min (~2 dynecm^−2^) to initiate neutrophil rolling. Phase contrast image was captured for 30 s at the centre of the microchannel every 0.5 s interval (total of 61 frames) at 20× magnification using MetaMorph software (Molecular Devices). To induce inflammation and disease conditions, DFF-purified neutrophils were also treated with tumor necrosis factor alpha (TNF-α, 10 ng/mL, Peprotech), PMA (2 nM, Sigma-Aldrich) or D-glucose (30 mM, Sigma-Aldrich) for 30 min at room temperature. The neutrophils were then washed at 1000× g for 4 min and resuspended to a concentration of ~10^6 ^cells/mL with 20 μM CaCl_2_ for rolling assay.

### Drug treatment of neutrophils

DFF-purified neutrophils were treated with pravastatin (20 μM, Merck) and metformin hydrochloride (1 mM, Sigma-Aldrich) for 1 h at room temperature. The neutrophils were then washed at 1000× g for 4 min and resuspended to a concentration of ~10^6^ cells/mL with 20 μM CaCl_2_ for rolling assay.

### Statistics

All numerical data were expressed as mean ± standard error (s.e.m.) unless specified otherwise. We assessed the statistical significance of the difference between two sets of data using Mann-Whitney test (unless specified otherwise) with *P* < 0.05 to be considered of significant difference. Linear regression was used to analyze associations between cell rolling phenotype (average rolling speed, specific speed frequency distribution) with neutrophil markers (PSGL-1, ROS, CD11b) or clinical measurements (HbA1c and lipid levels). All analysis was performed using GraphPad Prism V5.0 (GraphPad Software). Principle Component Analysis (PCA) was performed in the R language using the “ropls” package^61^. Missing values were inputted with mean value, and HbA1c value of 5.5 was assumed for healthy individuals. Prior to analysis, the data was mean-centered and scaled to unit-variance.

### Study approval

For all subjects, written informed consent was obtained during recruitment. All protocols were approved by the institutional review boards of Nanyang Technological University (IRB-2014-04-27) and Tan Tock Seng Hospital (2014/00416) and experiments were performed in accordance with relevant guidelines and regulations. A total of 32 subjects with Chinese or Indian ethnicity were recruited. For fingerprick blood sampling, blood was obtained from healthy donors using a disposable lancet (Roche Diagnostics Corp.) and collected in EDTA tubes (BD Microtainer). For blood sampling by venipuncture, ~3 mL of blood was collected into EDTA vacutainer (BD Biosciences) and shipped to NTU on the same day for microfluidics experiments and flow cytometry analysis.

*See Supplemental Information for more details on image processing and Giemsa staining.*

## Additional Information

**How to cite this article**: Hou, H. W. *et al*. Rapid and label-free microfluidic neutrophil purification and phenotyping in diabetes mellitus. *Sci. Rep.* **6**, 29410; doi: 10.1038/srep29410 (2016).

## Supplementary Material

Supplementary Information

## Figures and Tables

**Table 1:** Characteristics of healthy and T2DM patients.

Characteristics	T2DM (n = 16)	Healthy (n = 16)	P*
Age (Range)	56 (34–64)	34 (22–60)	–
CRP, mg/L	3.544 (1.123)	1.414 (0.659)	NS
HbA1c, %	9.544 (0.426)	n.a.	–
Fasting Glucose (mmol/L)	10.914 (1.130)	5.133 (0.116)	0.0001
Total-C (mmol/L)	4.244 (0.152)	4.914 (0.236)	NS
HDL-C (mmol/L)	1.094 (0.047)	1.328 (0.075)	0.03
LDL-C (mmol/L)	2.420 (0.139)	3.130 (0.229)	0.03
Triglyceride (mmol/L)	1.894 (0.420)	1.021 (0.217)	0.006

Average value shown with s.e.m. in parentheses, unless otherwise indicated. NS, Non-significant.
