# Supplementary material for: Rapid and label-free microfluidic neutrophil purification and phenotyping in diabetes mellitus
Source: Sci Rep. 2016 Jul 6;6:29410. doi: 10.1038/srep29410 (PMC4933935; doi:10.1038/srep29410)
Supplement: Supplementary Information [file srep29410-s1.pdf]

Rapid and label-free microfluidic neutrophil purification and phenotyping in diabetes mellitus

Han Wei Hou, Chayakorn Petchakup, Hui Min Tay, Zhi Yang Tam, Rinkoo Dalan, Daniel Ek Kwang Chew, King Ho Holden Li and Bernhard O. Boehm

Supplementary Data

Microdevice fabrication

The DFF spiral and straight channel microfluidic devices were fabricated in polydimethylsiloxane (PDMS) using standard soft lithography methods. Briefly, PDMS prepolymer was mixed in a 10:1 ratio (w/w) with curing agent and poured over the silicon wafers patterned with channel design. The PDMS mixtures were then cured at 80 °C for 2 h and peeled from the master template. Inlet and outlet holes (1.5mm) were punched using a biopsy puncher, and the devices were cleaned thoroughly with isopropanol before bonding to 1-mm-thick glass slides using air plasma machine (Harrick Plasma Cleaner).

Rolling velocity measurement

Rolling velocity measurement was done by image processing using MATLAB (Mathworks®). Briefly, image contrast was adjusted by top hat filtering to promote the contrast difference between cells and background. Next, contrast thresholding was applied for cell segmentation and background elimination. Blob analysis was used to detect and extract information (rough size and centroid position of cells) from segmented regions using 8-connected pixels criterion. The obtained information was used to eliminate non-cell component in the image such as leftover noise (small connected pixels) or un-eliminated background (large connected pixels). Then the centroid position of the segmented cells were matched between consecutive frames using K-nearest neighborhood to determine the distance traveled. Rolling velocity was calculated by dividing the distance by sampling time, which is 0.5 sec. For consistency, cells that appeared on the frame for less than 10 frames were discarded.

Neutrophil circularity

60x magnification images were used to determine the cell's circularity. The image contrast adjustment was similar as aforementioned section except watershed segmentation was introduced in between thresholding and blob analysis to segment the boundary of each cell. Area and perimeter of each cell from blob analysis were used to determine circularity as shown in the following equation.

$$Circularity = \frac{4\pi \times Area}{perimeter^2}$$

Giemsa Staining of Neutrophils

1-mm-thick glass slides were coated with poly-L-lysine (0.1 % (w/v) in H_2O, Sigma-Aldrich) for 5 min to promote cell adherence. The slides were left to dry before spotting ~10 μL of purified neutrophils (~10^6 cells/mL) on the coated slide. The cells were allowed to settle for 5 min and excess buffer was removed carefully before flooding with 1 – 2 mL of Wright-Giemsa (Sigma-Aldrich) stain solution for 2 min. The slides were rinsed with deionized water for another 1 min and air dried before viewing under a bright-field microscope.

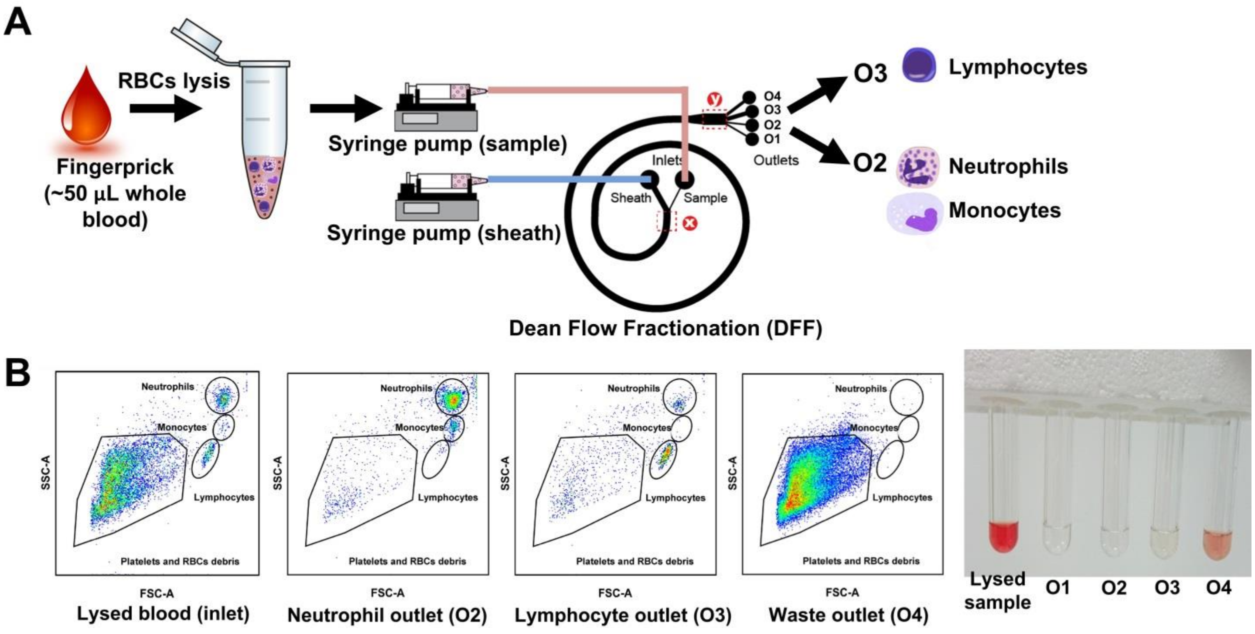

Figure S1 (**A**) Experimental setup of the DFF spiral device. After RBCs lysis, 2 syringe pumps are used for continuous perfusion of lysed blood samples and sheath buffer (1x PBS with 0.1% BSA) at different flow rates (1:10). (**B**) Representative flow cytometry analysis and clear solution of eluent from outlet 2 of device indicate efficient buffer exchange and separation of sorted neutrophils from lysed RBCs.

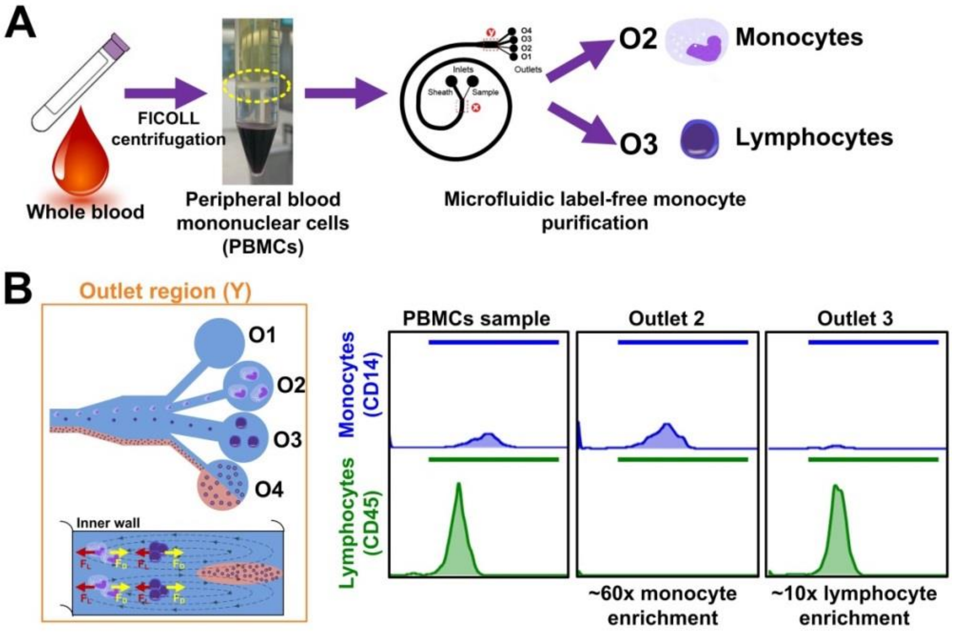

Figure S2 (**A**) Workflow for label-free fractionation of peripheral blood mononuclear cells (PBMCs) obtained from density centrifugation using Ficoll® Paque Plus. (**B**) Schematic illustration and flow cytometry analysis indicating efficient separation of monocytes and lymphocytes into outlet 2 and outlet 3, respectively, using DFF.

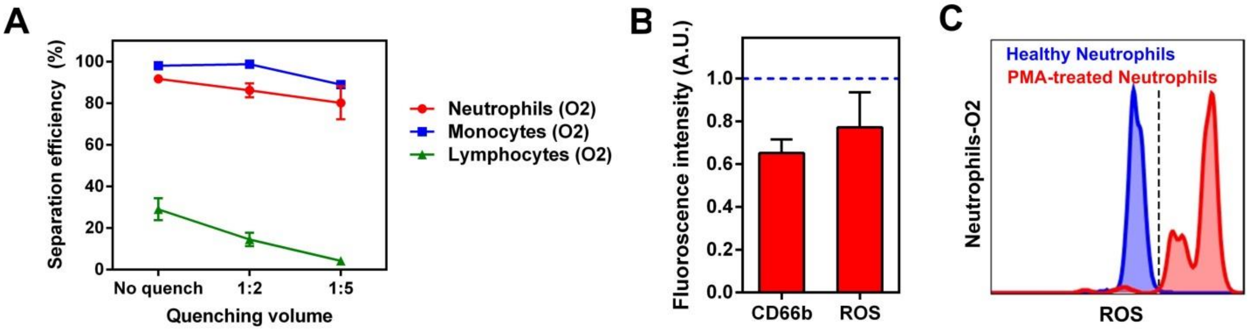

Figure S3 (**A**) Effect of quenching volume after RBCs lysis on leukocyte separation efficiency in outlet 2. A volume ratio of 1:5 (lysis buffer to quenching solution) is necessary for optimal neutrophil purification. (**B**) Slight decrease in fluorescence intensities of CD66b and intracellular reactive oxygen species (ROS) in DFF-sorted neutrophils indicate minimal activation of immune cells. Mean ± s.d. from n = 3. Dashed line (value of 1) represents normalized inlet signal. (**C**) Flow cytometry analysis of intracellular ROS level between sorted healthy and activated (PMA-treated) neutrophils.

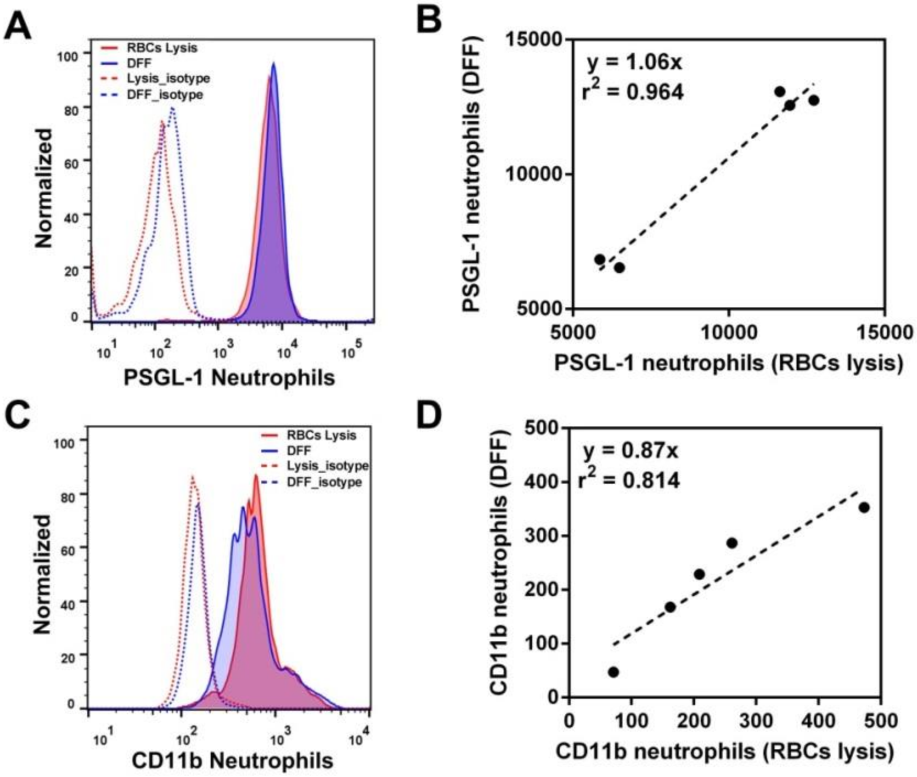

Figure S4 Flow cytometry analysis showing negligible differences in (**A, B**) PSGL-1 and (**C, D**) CD11b expression between DFF-sorted neutrophils and neutrophils washed with centrifugation after RBCs lysis.

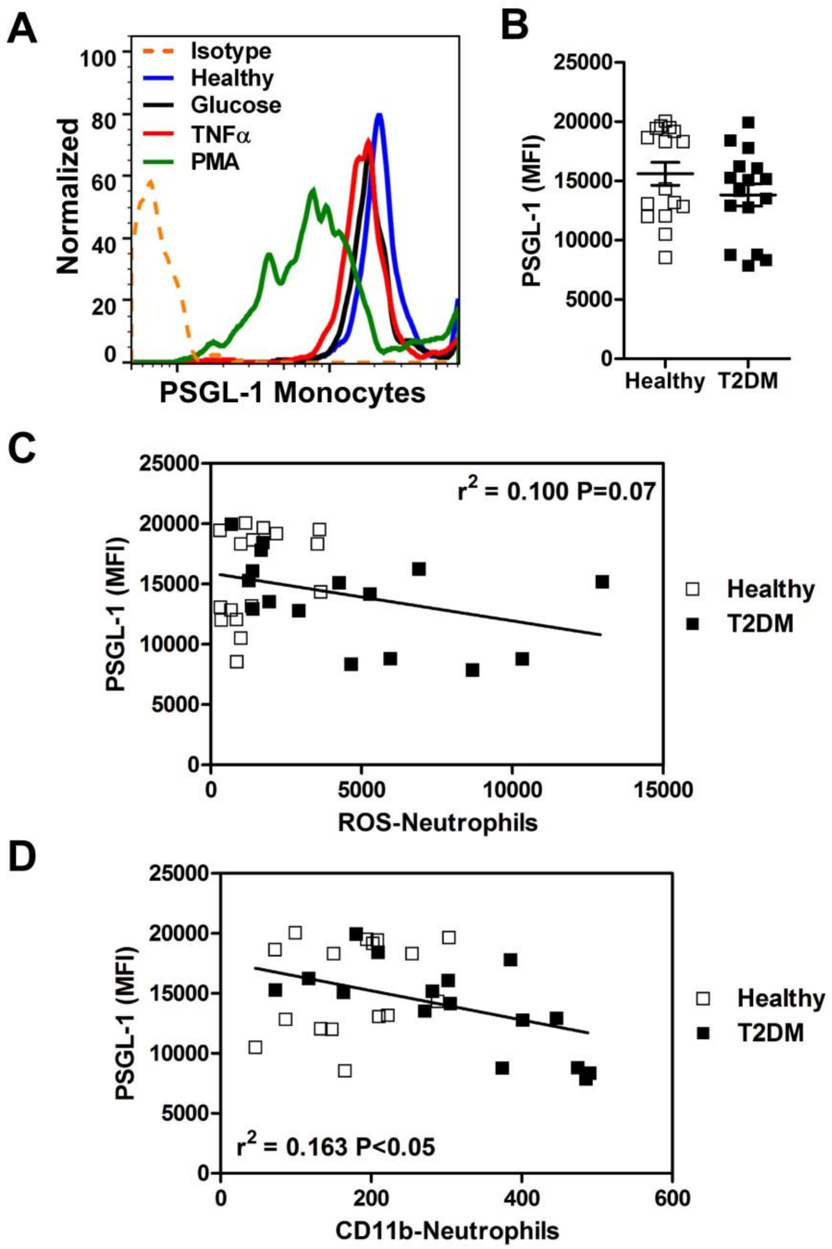

Figure S5 Flow cytometry analysis on (**A**) PSGL-1 expression on monocytes with different treatments. (**B**) PSGL-1 expression on monocytes from healthy (*n=16*) and T2DM patients (*n=16*). Mean ± s.e.m. Correlation of monocyte PSGL-1 level with (**C**) ROS and (**D**) CD11b expression on neutrophils. Data points are fitted with linear regression (solid line).

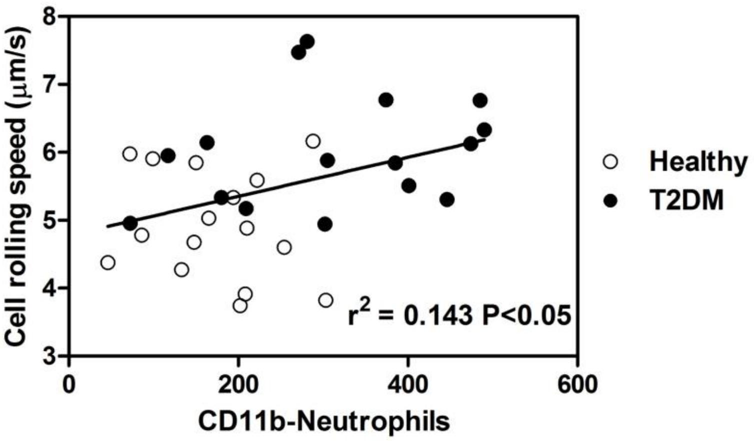

Figure S6 Association of average rolling speed with neutrophil CD11b expression in healthy and T2DM patients.

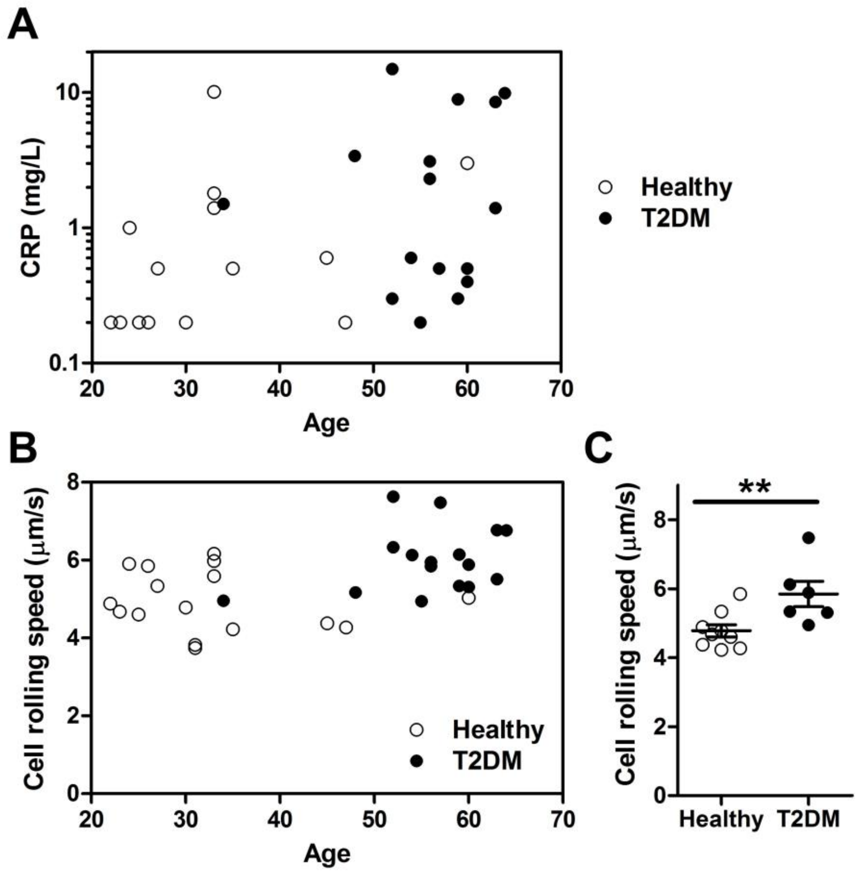

Figure S7 Association of patient age with (**A**) CRP level and (**B**) average neutrophil rolling speed in healthy and T2DM patients. (**C**) Average neutrophil rolling speed in subjects without inflammation (CRP<1mg/L).

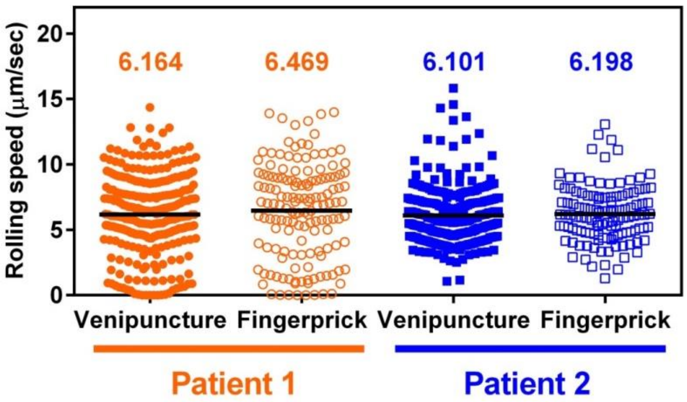

Figure S8 Non significant differences in neutrophil rolling speed between DFF-sorted neutrophils with blood obtained from venipuncture or fingerprick of the same patient.

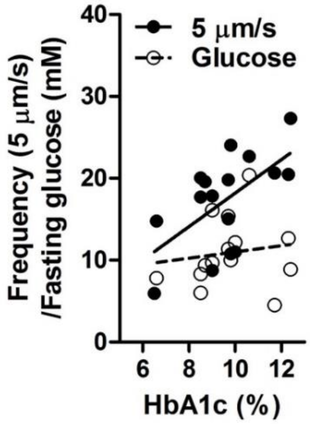

Figure S9 Dependence of rolling speed and fast glucose with HbA1c (%).

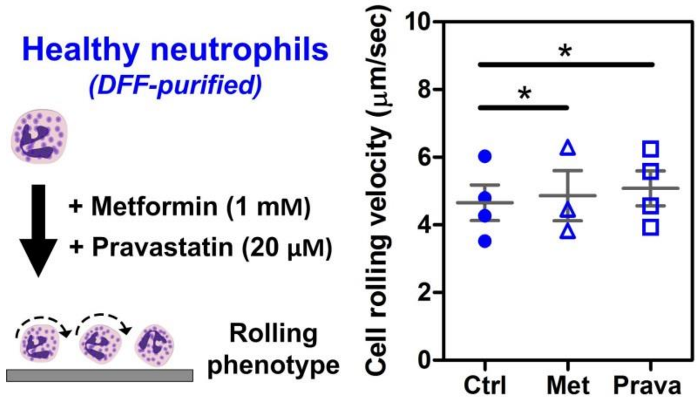

Figure S10 Average neutrophil rolling speed as a functional biomarker for drug assay. Higher rolling speed were observed in metformin (1mM) and pravastatin (20 μM)-treated neutrophils as compared to untreated healthy controls. Mean ± s.e.m n = 3-4. *$P < 0.05$ paired t-test.
